# Multi-modality imaging features of cavernous haemangioma of the right ventricle

**DOI:** 10.1093/ehjcr/ytae679

**Published:** 2024-12-23

**Authors:** Guangzong Su, Weiwei Wang, Yang Wu

**Affiliations:** Department of MRI, Wuhan Asia General Hospital, Wuhan 430050, China; Department of MRI, Wuhan Asia General Hospital, Wuhan 430050, China; Department of MRI, Wuhan Asia General Hospital, Wuhan 430050, China

**Figure:**
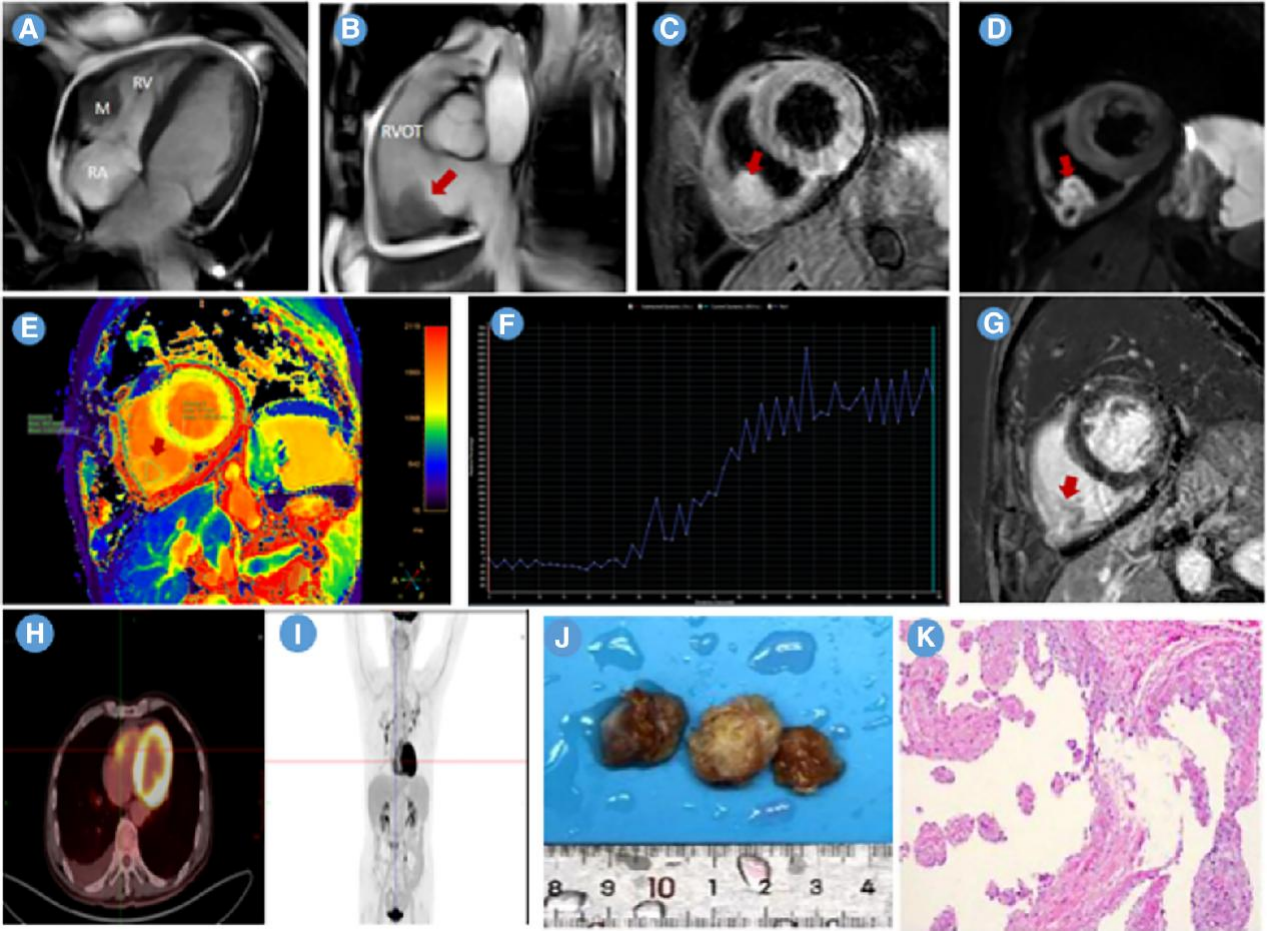


A 70-year-old male presented to the hospital with 5 days of unprovoked intermittent chest pain. Electrocardiogram showed pre-excitation syndrome. Bedside echocardiography demonstrated a mural hyperechoic mass in the right ventricle (RV). Coronary computed tomography angiography indicated that the coronary artery was normal. The cardiac magnetic resonance (CMR) imaging (*Panels A* and *B*; Supplementary data online, *Videos S1* and *S2*) revealed a wide sessile mass (3.0 × 2.5 cm) in the basal inferior and lateral wall of the RV with local muscular layer been invaded. The mass on the T2 weighted-short time of inversion recovery and diffusion-weighted imaging (*Panels C* and *D*) showed high signal. The native T1 value of tumour was 1638 ms (*Panel E*). The dynamic perfusion enhancement (*Panel F*; Supplementary data online, *Video S3*) and phase sensitive inversion recovery-delayed enhancement (*Panel G*) showed progressive hyperenhancement and obviously delayed hyperenhancement, which was similar to blood pool. The 18F-fluorodeoxyglucose positron emission tomography/computed tomography demonstrated a mild radioactive concentration in the mass (standard uptake value max 6.7) and no abnormal findings in other parts of the body (*Panels H* and *I*). Intraoperative findings are as follows: a sub-endocardial tumour with intact capsule and wide base infiltrated into the myocardium of the RV free wall. The gross specimens (*Panel J*) were fish-like texture. Histopathologic section (original magnification ×10; *Panel K*) confirmed it was a cavernous haemangioma.

Cardiac haemangioma is a rare benign cardiac tumour in the RV. Surgical resection is the first choice for patients with obvious symptoms. Preoperative multi-modality imaging can determine the mass size, location, and relationship with anatomical structures, while the pathognomonic signs detected by CMR are extremely valuable for pre-surgical diagnosis.

## Supplementary Material

ytae679_Supplementary_Data

## Data Availability

The data underlying this manuscript will be shared on reasonable request to the corresponding author.

## Notes

**Consent:** The authors confirm that written consent has been obtained from the patient for the submission and publication of this cardiovascular flashlight, including images and associated text, in accordance with the COPE guidelines.

**Funding:** None declared.

